# Personal

**Published:** 1909-05

**Authors:** 


### PERSONAL.

Dr. Roswell Park announces the removal of his offices from 510 Delaware avenue to 430 Delaware avenue. Dr. Edgar R. McGuire has removed his office and residence to the same address. The office hours of Drs. Park and McGuire will be from 2 to 4 P. M., Sundays by appointment only. Telephones: Bell, Tupper 4; Frontier, 637.

Dr. Harry R. Trick, of Buffalo, announces his removal from No. 1195 Main street to No. 605 Elmwood avenue, "Elmwood Heights," apartment No. 1.

Dr. James E. King, of Buffalo, has succeeded Dr. DeWitt G. Wilcox in the conduct of Lexington Heights Hospital, 173 Lexington Avenue, Buffalo, N. Y.

Dr. George N. Jack, of Buffalo, announces the removal of his office from 91 Niagara street to 901 Mutual Life Building, 202-218 Pearl street. Hours: 9 to 1. Excepting Sundays.

Dr. Lucien Howe, of Buffalo, who has been in attendance upon the International Ophthalmological Congress at Naples, has returned. He was accompanied by Mrs. Howe.

Dr. W. L. Allen, of Williamsville, has been elected president of the Board of Health of that village for the current year. Dr. W. H. Baker is appointed health officer for the year.

